# Efficacy and safety of self-made covered coronary stent in the treatment of coronary artery perforation

**DOI:** 10.1186/s12872-023-03575-3

**Published:** 2023-11-04

**Authors:** Wen An, Jian Ye, Bingyu Han, Xu Wang, Chao Han, Junqing Gao, Zongjun Liu

**Affiliations:** 1 Department of Cardiology, Putuo Hospital, Shanghai University of Traditional Chinese Medicine, Shanghai, 200062 People’s Republic of China; 2 Department of Cardiology, The Affiliated Hospital of Shaoxing University, Shaoxing, People’s Republic of China

**Keywords:** Coronary covered stent, Coronary perforation, Thrombus, CaIMR

## Abstract

**Objective:**

To observe the efficacy and safety of self-made covered coronary stent in the treatment of coronary artery perforation.

**Methods:**

Covered coronary stent was prepared by wrapping 3 M film on the surface of coronary stents. The beagle dogs were divided into control group and experimental group. A drug-eluting stent (DES) was implanted in the control group. The covered stent was applied to block the coronary branches of beagle dogs. The CaIMR value after stent placement was calculated by FlashAngio software. The effect of blocking the coronary branches on blood flow was observed by coronary angiography (CAG). The condition of the implanted stent was observed by optical coherence tomography (OCT), and the histopathologic examination of the coronary vessel implanted stent was performed by HE staining.

**Results:**

The best number of layers was 2. Compared with the control group, the CaIMR of the experimental group increased (*p* < 0.05). A lot of in-stent thrombosis were found in the experimental group and obvious blood flow obstruction during follow-up. HE staining showed that stents implanted in the two groups adhered well to the wall of the blood vessel, but in-stent thrombosis and intimal hyperplasia were founded in the experimental group, while the in-stent restenosis was not founded.

**Conclusion:**

The self-made coronary covered stent can effectively block the leakage caused by coronary perforation, but the stent endothelialization is poor, which easily causes stent thrombosis and restenosis, so it is not recommended as a routine remedy.

## Foreword

Percutaneous coronary intervention(PCI) can rapidly improve myocardial ischemia in patients, and is still one of the optimal treatments for coronary heart disease. However, complications may occur during PCI, especially in complicated lesions such as bifurcation lesions, chronic total occlusion (CTO) and severe calcification lesions. Coronary artery perforation is a common and quite serious complication of CTO-PCI with high mortality [1]. Timely detection of perforation and occlusion can reduce the mortality of patients. Usually, balloon occlusion or heparin antagonist were used to treat coronary artery perforation. However, if the perforation can not be occluded, the clinician will usually seek help from covered stents [2]. Due to the fact that in China and some regions, covered stents are not routinely prepared in the catheter laboratory and sometimes may not be suitable for remedial treatment of ruptured coronary intervention due to size mismatch. Therefore, in clinical practice, interventional doctors will use self-made covered stents for emergency treatment of coronary artery rupture. At present, there is no uniform standard for the self-made covered stent, and no systematic evaluation of the efficacy. Therefore,this study observed the expansion of the self-made covered stent in vitro to find the best number layers of film. According to the results of in vitro release, a suitable covered stent was made to occlude the small arteries of experimental dogs to observe the efficacy and safety of self-made simple covered stent in the treatment of coronary artery perforation.

## Materials and methods

### Experimental animals

A total of 20 adult beagles, male or female, weighing 20 ~ 25 kg, were purchased from Shanghai Jiagan Biotechnology Co., Ltd. Using the random number method [3], the experimental animals were randomly divided into experimental group and control group with 10 animals in each group. All beagle dogs were fasted for 24 h and given a loading dose of aspirin (300 mg) and clopidogrel (300 mg) before the beginning of the experiment, then given a maintenance dose of aspirin (100 mg) and clopidogrel (75 mg) postoperatively every day. This study was performed according to the National Institutes of Health Guide for the Care and Use of Laboratory Animals and ARRIVE guidelines, and was approved by the Animal Ethics Committee of Putuo Hospital Affiliated to Shanghai University of Traditional Chinese Medicine.

### Fabrication method of covered stent

An appropriate length of 3 M™ Tegaderm™ transparent film dressing (frame type 1624 W, 3 M Company, USA) was cut according to the length of stent ( which was usually 2 mm shorter than the stent), this film formed the outer layer of the self-made covered stent, and the cut transparent film was then wrapped around the surface of the stent(the parts of the stent that are not covered at both ends should be of the same length) [4], which was a self-made covered stent. In this experiment, the stent was wrapped 1–5 layers with film, and the expansion of the stent of wrapping different number layers with the film in vitro was observed to select the best number of layers, and then according to the best number of layers a covered stent was made for animal experiments. The stents used in this study were all drug-eluting stents(DES)(firehawk, MicroPort Medical, Shanghai).

### Stent implantation

Induced anesthesia for experimental animals were used with tiramine (4 mg/kg), diazepam (1 mg/kg), and xylazine (2.5 mg/kg) intramuscularly and propofol (3 mg/kg) intravenously, and then propofol (10 mg•kg-1•h-1) was continuously infused by intravenously. Electrocardiogram of the animals was continuously monitored, and endotracheal intubation for the animals was performed to support mechanical ventilation. A 6 F sheath was inserted into the right femoral artery and heparin was injected (4,000 IU) through the sheath to prevent thromboembolism. A Medtronic 6 F left-guided catheter was used to coronary angiography, and then the stents were implanted in the middle and distal segments of the left circumflex branch (LCX) under the guidance of coronary angiography. All stents were implanted with a stent/artery (diameter) ratio of 1.1–1.2:1, length 20-38 mm. A DES was implanted in the control group. In the experimental group the self-made covered stent with the best number of layers according to the results of release in vitro, was implanted to cover the ostium of the side branch of the coronary artery. When the stent was fully inflated and attached to the wall of the vessel, no blood flow in the small side branch was observed, which indicated that the ostium of the small side branch was fully blocked. Coronary angiography was performed 1 month after operation.

### Coronary angiography-derived microvascular resistance index (CaIMR)

CaIMR was calculated immediately after stent implantation using CPFD-IMR measurement software (FlashAngio, Rainmed Ltd., Suzhou, China). Briefly, after stent implantation, nitroglycerin(0.1 mg) is injected into the coronary artery, and after exposure for 1s, the contrast agent was injected into the coronary arteries at a rate of 4 ml/s, obtain the aortic pressure wave and coronary angiograms from two projections, CaIMR is then performed using the following formula. The method described by Hu Ai et al. [5] was used for CaIMR determination.

$$\mathrm{CaIMR}\;=\;{\left({\mathrm P}_{\mathrm d}\right)}_{\mathrm{hyp}}\cdot\mathrm L/\left(\mathrm K\cdot{\mathrm V}_{\mathrm{diastole}}\right)$$

where L is a constant (non-dimentional) that mimics the length from the inlet to the distal position (L = 75, mimicking 75 mm downstream from the inlet of coronary arterial tree), (P_d_)_hyp_ is the mean pressure (unit: mmHg) at the distal position at the maximal hyperemia, V_diastole_ is the mean flow velocity (unit: mm/s) at the distal position at diastole, and K is a constant (K = 2.1) and V_hyp_ = K⋅V_diastole_ refers to the mean flow velocity (unit: mm/s) at the distal position at the maximal hyperemia.

### Optical coherence tomography (OCT)

OCT was performed using a disposable intravascular imaging catheter (C408644, Abbott Medical Products, Inc., USA). The catheter was sent to the distal end of the stent, and the catheter was automatically pulled back at a speed of 20 mm/s. When the catheter was automatically pulled back, contrast agent was injected with a syringe at a rate of 2–5 mL/s.The stent area was assessed by OCT immediately after stent placement and 1 month later.

### Histopathological examination

The animals were euthanized by injecting pentobarbital (100 mg/Kg) in batches 1 month and 3 months after the operation. The blood vessels of the stent segments were taken out and embedded in hard tissue resin. The endothelial conditions and restenosis of the stents were observed by HE staining.

### Statistical analysis

SPSS 22.0 statistical software(IBM Corp. IBM SPSS Statistics for Windows, Version 22.0. Armonk, NY: IBM Corp) was used for data processing, and the measurement data were expressed as mean ± standard deviation. The comparison of normal distribution and variance homogeneity of measurement data were conducted by variance analysis, and the deviation measurement data was made by nonparametric MannWhitney U test. The counting data were expressed as frequency or percentage. The χ^2^ test or Fisher exact probability method were used to compare the non-grade data between groups, and both of them used two-sided test, with *P* < 0.05 as significant difference.

## Results

### Preparation of covered stent

**Fig. 1:**
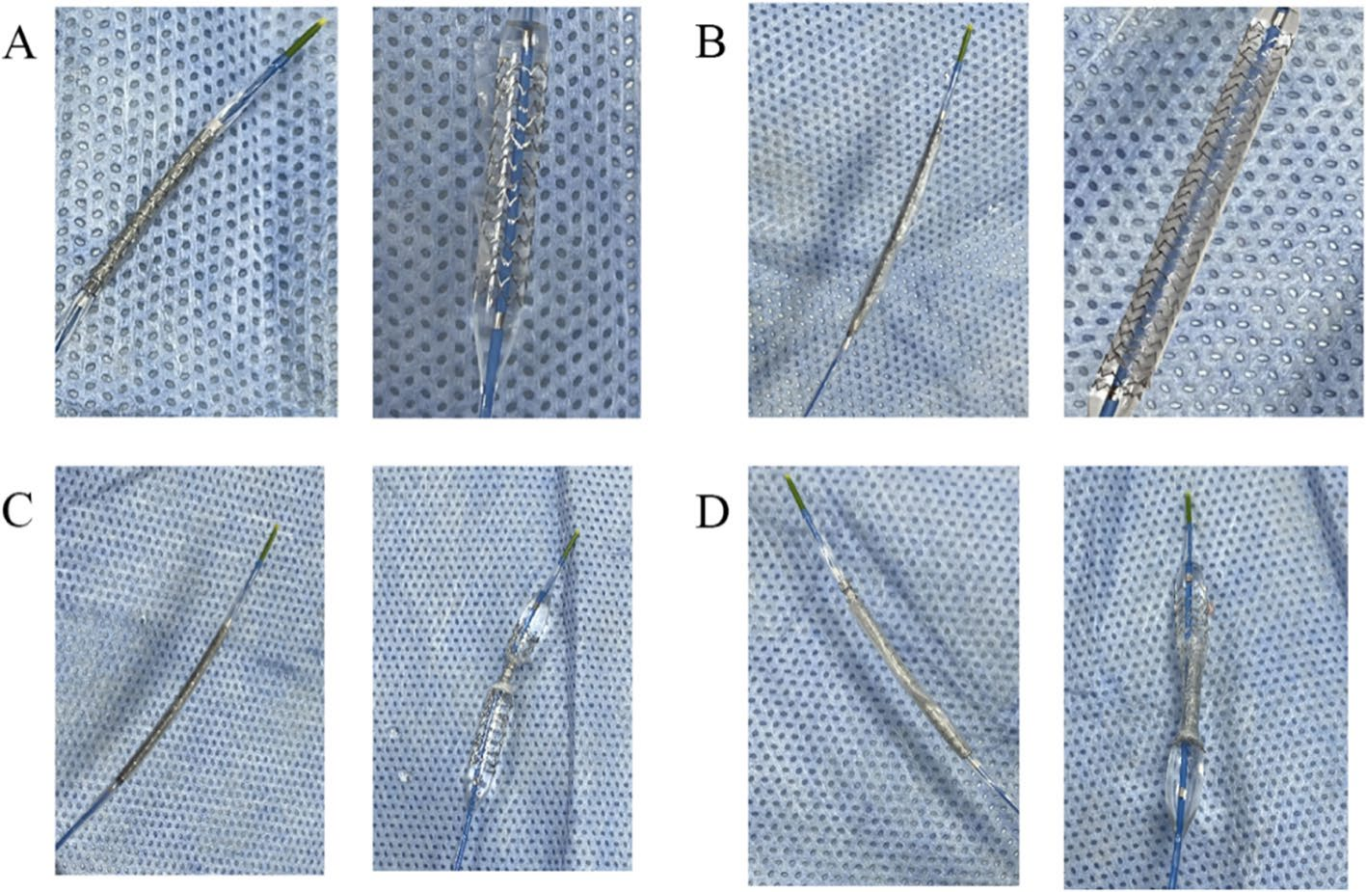
Shows the expansion diagram of the covered stent. **A** In the case of 1-layer, the stent film falls off after expansion; **B** In the case of 2-layer, the stent is fully expanded during the naming pressure; **C** In the case of 3-layer, the stent cannot be complete expansion under the naming pressure; **D** In the case of 4-layer, the stent cannot be fully expanded under the named pressure, and the stent is deformed by increasing the pressure

The results of expansion of the self-made covered stent at named pressure in vitro test are shown in Table 1. It is suggested that 2 layers are the most suitable winding turns (Fig. 1B). Too few layers will cause the 3 M film to collapse (Fig. 1A), and too many layers will lead to the failure of stent expansion. As the pressure increased, some stents were prone to the phenomenon of film retraction together (Fig. 1C). In addition, an excessive number of layers may result in the insufficient expansion or the failure of the stent, even though the inflation pressure is high enough to the burst pressure (Fig. 1D).

**Table 1:** Expansion of covered stents at named pressures

Inner diameter	2.5mm	2.75mm	3.0mm	3.5mm	4.0mm
layers					
1	collapse	collapse	collapse	collapse	collapse
2	Normal	Normal	Normal	Normal	Normal
3	Normal	Normal	Unexpanded	Unexpanded	Unexpanded
4	Unexpanded	Unexpanded	Unexpanded	Unexpanded	Unexpanded
5	Unexpanded	Unexpanded	Unexpanded	Unexpanded	Unexpanded

### Coronary angiography

**Fig. 2:**
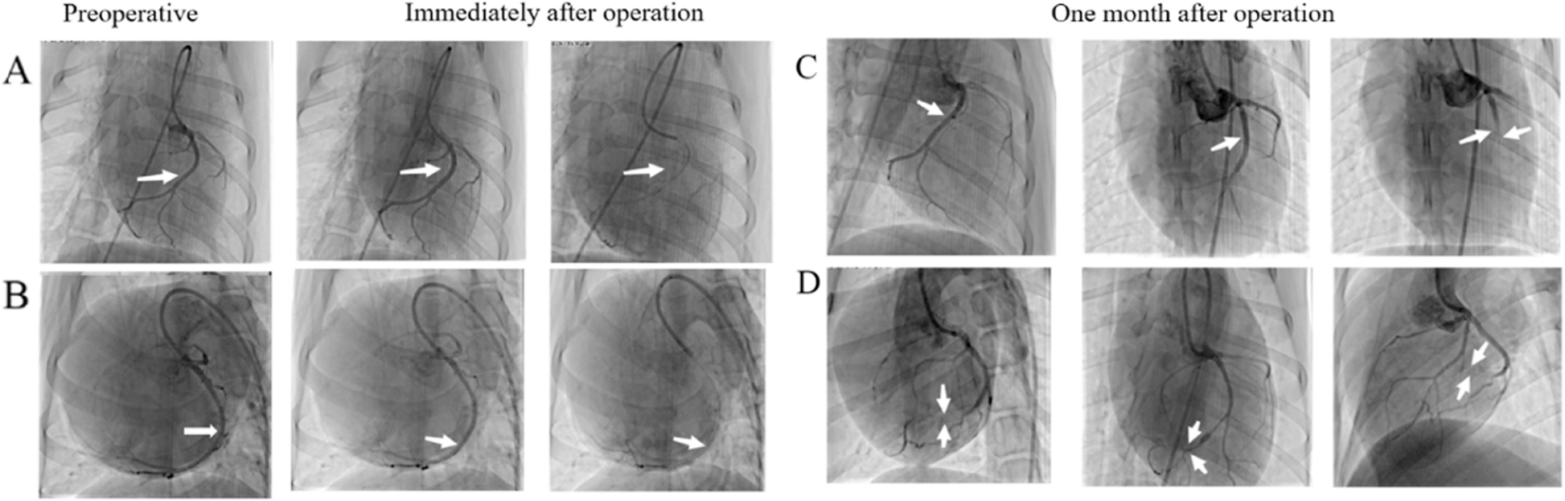
Shows coronary angiography.  **A** Comparison before and after operation in the control group; **B** Comparison before and after operation in the experimental group, showing complete occlusion of small sub-branch; **C** In the control group, no blood flow obstruction was found during fellow-up; **D** In the experimental group, the stent stenosis, blood flow occlusion and formation of collateral circulation were found in 1 month later.  The arrow in the figure is the stent placement site

According to the results of in vitro expansion, film was used to wrap the stent with 2 layers in animal experiments. Immediately after the stent implanted, the blood flow of the branch blood vessels in the experimental group disappeared completely (Fig. 2B), while the blood flow of the control group was not affected (Fig. 2A). Coronary angiography was repeated 1 month later, no animals died unexpectedly in the study, and all stent placement sites in the experimental group had blood flow occlusion (Fig. 2D), while there was no obvious blood flow obstruction in the control group (Fig. 2C).

### CaIMR

**Fig. 3:**
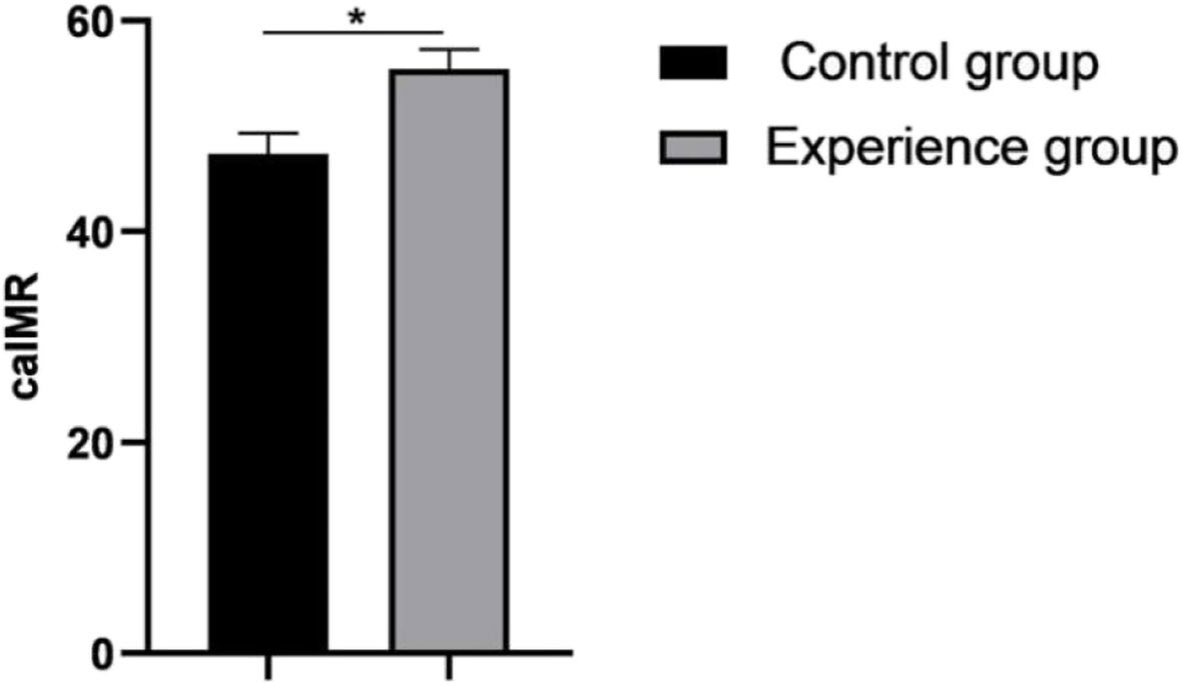
Shows the comparison of CaIMR between the two groups, compared with the control group, *P<0.05

The CaIMR was measured immediately after stent implanted (Fig. 3). Compared with the control group (47.36 ± 1.95), the CaIMR of the experimental group (55.42 ± 1.88) was significantly higher (*P* < 0.05).

### OCT

**Fig. 4:**
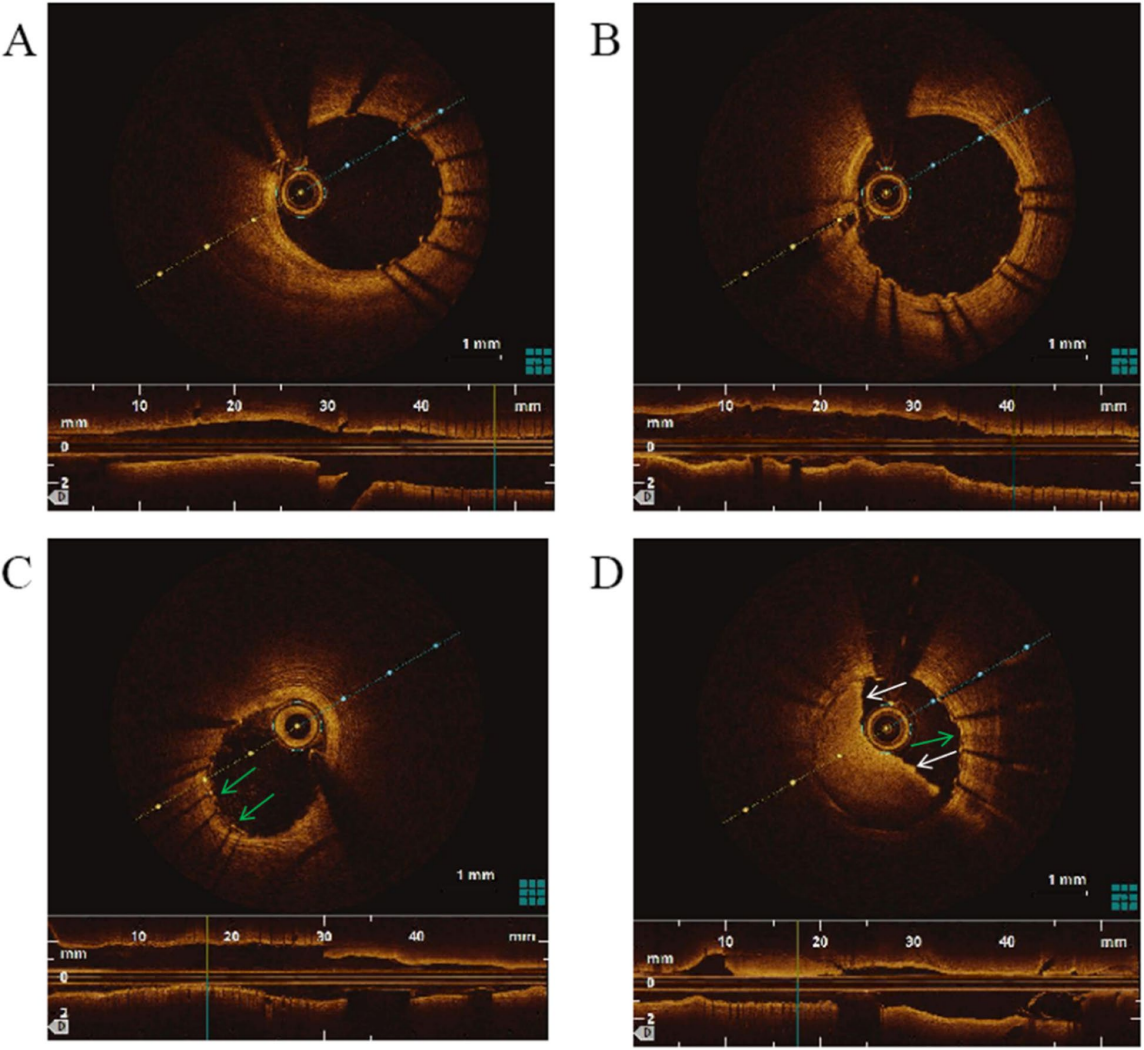
Is an OCT image. **A** immediately after stenting in the control group; **B** 1 month after stenting in the control group, uniform coverage of the stent surface was observed; **C** immediately after stenting in the experimental group, film could be observed ; **D** 1 month after stenting in the experimental group, the film and a large number of thrombus can be observed in the stent. The green arrow is the film, and the white arrow is the thrombus

OCT was performed after stent implanted, which showed that stents in two group adhered well (Fig. 4A); and the 3 M film was visible in experimental group (Fig. 4C). OCT was reexamined 1 month after operation (coronary artery was required to be opened in the experimental group). Uniform coverage of the stent surface was observed in the control group (Fig. 4B), while the stent in the experimental group had a large number of thrombus (Fig. 4D).

### HE staining

**Fig. 5:**
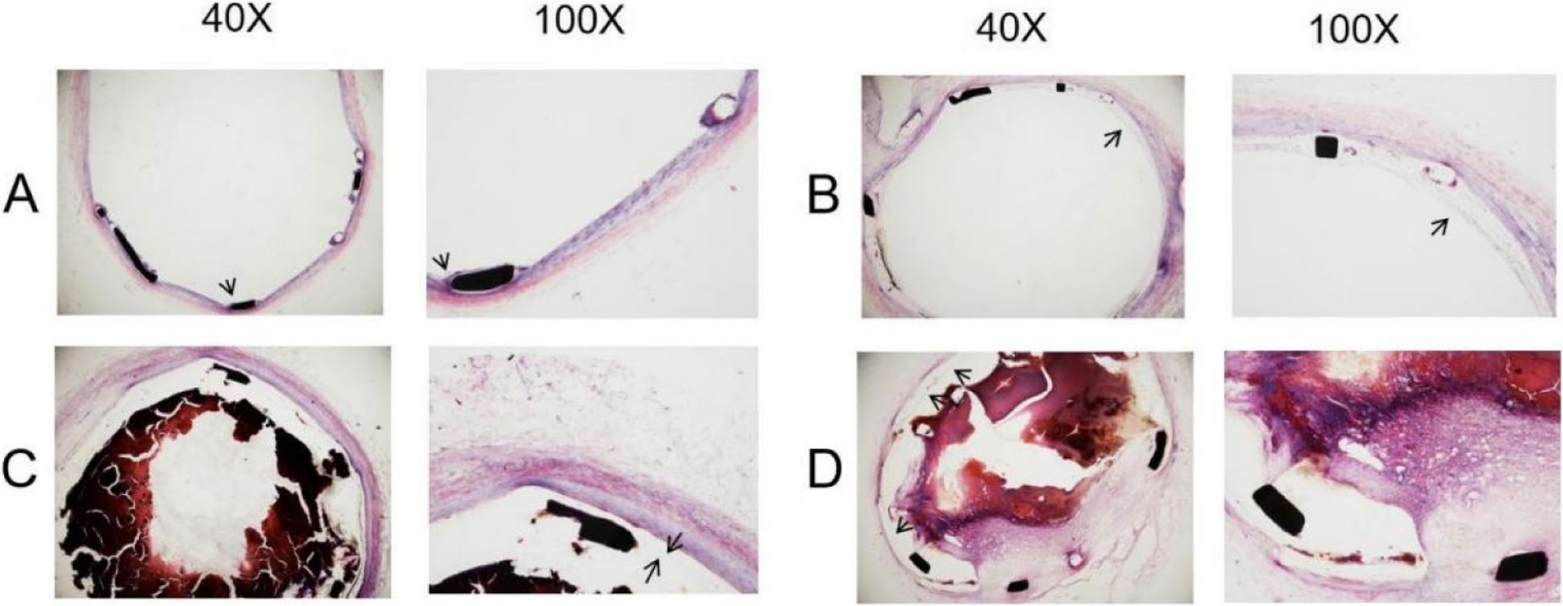
Shows HE staining. **A** 1 month after operation in control group, **B** 3 months after operation in control group, **C** 1 month after operation in experimental group, **D** 3 months after operation in experimental group. The **A** and **B** arrowheads were endothelium, and the **C** and **D** arrowheads were 3M film. In the control group, the excessive neointimal proliferation or thrombosis was not found.;but in the experimental group,large amounts of thrombosis were observed at 1 month and 3months; Significant excessive neointimal proliferation,inflammatory cell infiltration, lipid deposition and myometrial collagen fiber degeneration can be observed at 3 months

At 1 month and 3 months after stent implanted, the blood vessels in the stent area were cut out for HE staining. At 1 month, the stent was partially endothelialized in the control group (Fig. 5A), the film and a large number of thrombus were seen in the experimental group (Fig. 5C). At 3 month, almost completely endothelialized in the control group (Fig. 5B), but significant intimal hyperplasia and thrombus were observed in the experimental group (Fig. 5D).

## Discussion

With the development of interventional techniques and strategies, PCI-related complications have been greatly reduced, but in the treatment of complex lesions, such as bifurcation lesions, CTO and so on, the incidence of complications is still high [1]. Coronary artery perforation is one of the major complications of PCI [6, 7]. According to a survey of interventional cardiologists, coronary artery perforation is still a PCI-related complication that they are concerned about [8]. A significant proportion of patients with coronary artery perforation develop pericardial tamponade, which affects hemodynamics, and has a severe prognosis [2, 6, 9].

Coronary perforation can be divided into three types according to the Ellis classification as follows: class-I points out an extraluminal crater without extravasation; class II points out pericardial/myocardial blush without contrast jet extravasation; class III points out the continuous outflow of contrast agent through perforation or injection into the anatomical cavity [10]. Typically, when a coronary perforation is found, the first approach is balloon occlusion [11], that is, the gap is closed by expanding the balloon. When the balloon tamponade fails, small blood vessels such as collateral vessels can be embolized by materials, including subcutaneous fat, autologous thrombus, polyvinyl alcohol particles, gelatin sponges and microspheres, etc., all of which can form blood clots, thus closing the gap [11, 12]. However, material embolization can not solve the problem of the perforation of the major vessels, such as the main blood vessel or the branch blood vessel, so it is necessary to rely on the covered stent or surgery to stop bleeding.The covered stent is one of the most commonly used methods [2]. Covered stent can block the gap, but the incidence of short-term and long-term adverse events increases significantly [13]. In reality, covered stents are not prepared in many catheter laboratories, so that cardiac interventional doctors may have to make covered stent on-site.

The film covering the surface of the stent is still the most common method of making simple covered stent [4], but there is no unified standard. The results of the vitro experiments showed that wrapping two layers of film around the surface of the stent was the most appropriate, and the film was easy to fall off when the number of thin film layers was too small. Nonetheless, too many covering layers (4–5 layers) might cause the stent to fail to expand or even deform. In vivo experiments, we successfully occluded branch vessels with a 2-layer covered stent, but during the follow-up, it could be found that stent thrombosis or restenosis events occurred with the covered stent, even with the use of dual anti-platelet; This suggested that the safe use of simple membrane-covered stent was worth considering. Previous studies have found that self-made cover stent may cause thrombosis and obstruction instantly [14], in our study ,in OCT images, a free membrane between the sections, affecting the hemodynamics, which was a possible cause of stent thrombosis, was observed. In addition, we observed from HE staining that the covered stent was unable to be endothelialized, whereas DES endothelialization was more intact, and poor endothelialization was also an adverse factor for stent thrombosis and restenosis [15, 16]. After the covered stent is implanted, the endothelium will be completely “Covered” by the covered stent, which affects endothelial and vascular elastic function, and a gap between the film and stent, which can lead to slow blood flow, aggregation of red blood cells and platelets to thrombosis. At the same time, sustained rejection led to excessive inflammation at the stent implantation site, coupled with the membrane blocking the effect of DES on inhibiting smooth muscle proliferation, so we saw stent intimal hyperplasia after 3 months. Previous studies have suggested that the use of anticoagulants may reduce covered stent thrombosis, but it can not be completely avoided [17].

CaIMR, developed from IMR, can complete the evaluation of microcirculation function without using pressure guide wire after coronary angiography. It is currently believed that CaIMR can have the same evaluation effect as IMR [5]. Higher IMR or CaIMR values tend to mean a worse prognosis [5, 18–20]. In this study, the postoperative CaIMR was significantly higher in the experimental group than in the control group, suggesting a worse prognosis in the experimental group, which was also confirmed during the follow-up (stent thrombosis or restenosis was common in the experimental group). In addition, in clinical overlapping stents may also have an adverse effect on the prognosis of patients with complex lesions, which often require multiple stents implanted [21], it means that we should minimize the use of covered stent.

## Conclusion

In this study, we found that the temporary simple coronary covered stent can effectively block the perforation of coronary artery, and the optimal number of covering circles of the film is 2. However, it should be noted that the in-stent thrombosis/restenosis caused by temporary self-made covered stent is almost inevitable. In conclusion, our study suggests that temporary self-made simple covered stents should be avoided as much as possible, and that anticoagulation should be intensified when it is used to reduce the problem of in-stent thrombosis/restenosis.

## Limitations and future perspective

This study only looked at the differences between DES and self-made covered stents, so the limitation of this study is that there is no comparison between self-made covered stents and other methods of occlusion of coronary artery perforation. However, the results of this study still suggest that the use of self-made covered stents should be reduced.

## Data Availability

The datasets used and analyzed during the current study are available from the corresponding author on reasonable request.

## References

[1] Hirai T, Grantham JA, Perforation, Mechanisms (2021). Risk stratification, and management in the Post-coronary Artery Bypass Grafting Patient[J]. Interv Cardiol Clin.

[2] Cerrato E, Pavani M, Barbero U (2021). Incidence, Management, Immediate and Long-Term Outcome of Guidewire and device related Grade III coronary perforations (from G3CAP - cardiogroup VI Registry)[J]. Am J Cardiol.

[3] Garg Y, Kakria N, Katoch CDS, Bhattacharyya D (2020). Exhaled nitric oxide as a guiding tool for bronchial Asthma: a randomised controlled trial. Med J Armed Forces India.

[4] Song X, Qin Q, Chang S et al. Clinical outcomes of self-made polyurethane-covered stent implantation for the treatment of coronary artery Perforations. J Interv Cardiol,2021;2021:6661763. 10.1155/2021/6661763 PMC8143889 34104120

[5] Ai H, Feng Y, Gong Y (2020). Coronary angiography-derived index of Microvascular Resistance. Front Physiol.

[6] Bauer T, Boeder N, Nef HM (2015). Fate of Patients With Coronary Perforation Complicating Percutaneous Coronary Intervention (from the Euro Heart Survey Percutaneous Coronary Intervention Registry)[J]. Am J Cardiol.

[7] Riley RF, Sapontis J, Kirtane AJ (2018). Prevalence, predictors, and health status implications of periprocedural complications during coronary chronic total occlusion angioplasty[J]. EuroIntervention,2018.

[8] Simsek B, Kostantinis S, Karacsonyi J (2022). International percutaneous coronary intervention complication survey. Catheter Cardiovasc Interv.

[9] Kassier A, Fischell TA. Managing coronary artery perforation after percutaneous coronary intervention. Expert Rev Cardiovasc Ther. 2022;20(3):215–22. 10.1080/14779072.2022.2059465 35341445

[10] Ellis SG, Ajluni S, Arnold AZ (1994). Increased coronary perforation in the new device era. Incidence, classification, management, and outcome. Circulation.

[11] Jacob D, Savage MP, Fischman DL (2022). Novel approaches to coronary perforations: everything but the Kitchen Sink[J]. JACC Case Rep.

[12] Abdalwahab A, Farag M, Brilakis ES (2021). Management of coronary artery Perforation[J]. Cardiovasc Revasc Med.

[13] Pavani M, Cerrato E, Latib A (2018). Acute and long-term outcomes after polytetrafluoroethylene or pericardium covered stenting for grade 3 coronary artery perforations: insights from G3-CAP registry. Catheter Cardiovasc Interv.

[14] Lee W-C, Hsueh S-K, Fang C-Y (2016). Clinical outcomes following covered stent for the treatment of coronary artery perforation. J Interv Cardiol.

[15] Scafa UA, Niculescu AG, Grumezescu AM (2021). Cardiovascular Stents: A Review of Past, Current, and Emerging Devices[J]. Materials (Basel).

[16] Hao D, Fan Y, Xiao W (2020). Rapid endothelialization of small diameter vascular grafts by a bioactive integrin-binding ligand specifically targeting endothelial progenitor cells and endothelial cells[J]. Acta Biomater.

[17] Chen Y (2023). Covered stent treatment for arterial Complications after pancreatic Surgery: risk assessment for recurrence and peri-stent implantation management. Eur Radiol.

[18] Lee JM, Choi KH, Choi JO (2021). Coronary Microcirculatory Dysfunction and Acute Cellular Rejection After Heart Transplantation[J]. Circulation.

[19] Noirclerc N, Marliere S, Bakhti A (2022). Impact of a micro-net mesh technology covering stent on coronary microvascular dysfunction in patients with high thrombus burden[J]. Catheter Cardiovasc Interv.

[20] Abdu FA, Liu L, Mohammed AQ (2021). Prognostic impact of coronary microvascular dysfunction in patients with Myocardial Infarction with non-obstructive coronary arteries[J]. Eur J Intern Med.

[21] Şaylık F, Çınar T, Selçuk M (2023). Comparison of outcomes between single long stent and overlapping stents: a meta-analysis of the literature. Herz.

